# Molecular analysis of methicillin-resistant *Staphylococcus aureus* isolates from four teaching hospitals in Iran: the emergence of novel MRSA clones

**DOI:** 10.1186/s13756-020-00777-8

**Published:** 2020-07-17

**Authors:** Farzaneh Firoozeh, Mitra Omidi, Mahmood Saffari, Hossein Sedaghat, Mohammad Zibaei

**Affiliations:** 1 Department of Microbiology, School of Medicine, Kashan University of Medical Sciences, Kashan, Iran; 2 Department of Microbiology, School of Medicine, Alborz University of Medical Sciences, Karaj, Iran; 3 Dietary Supplements and Probiotic Research Center, Alborz University of Medical Sciences, Karaj, Iran; 4 Evidence-based Phytotherapy & Complementary Medicine Research Center, Alborz University of Medical Sciences, Karaj, Iran; 5 Department of Parasitology and Mycology, School of Medicine, Alborz University of Medical Sciences, Karaj, Iran

**Keywords:** MRSA, MLST, SCC*mec* typing, MSCRAMMs, Genotyping

## Abstract

**Background:**

The global spread of methicillin-resistant *Staphylococcus aureus* (MRSA) infections necessitates the use of validated methods for the identification and typing of this bacterium. This study aimed to determine the distribution of main molecular types of MRSA strain circulating among hospitalized patients in teaching hospitals in Isfahan and Kashan.

**Methods:**

A total of 146 *Staphylococcus aureus* strains were isolated from patients in four teaching hospitals in Isfahan and Kashan during June 2017 to September 2018. The antimicrobial resistance patterns of *Staphylococcus aureus* strains were performed by disc diffusion method. The MRSA strains were identified phenotypically and confirmed by PCR assay. The prevalence of microbial surface components recognizing adhesive matrix molecules (MSCRAMMs) genes among MRSA strains was evaluated by multiplex PCR. The genotypes of MRSA strains were determined by multilocus sequence typing and SCC*mec* typing.

**Results:**

Of 146 *Staphylococcus aureus* isolates, 24 (16.4%) isolates were identified as MRSA strains. According to antimicrobial susceptibility testing the highest resistance rates were seen for tetracycline, erythromycin, ciprofloxacin and gentamicin. All of *Staphylococcus aureus* isolates were susceptible to vancomycin whereas 3 (2.1%) isolates were resistant to linezolid. Three different SCC*mec* types were obtained among MRSA strains including 16 (66.7%) SCC*mec* type V, 3 (12.5%) SCC*mec* type III and 5 (20.8%) SCC*mec* type II. Of 24 MRSA isolates 20 (83.3%) carried MSCRAMMs genes including *eno* (70.8%), *fib* (54.1%)*, cna* (25.0%), *fnbB* (16.6%)*, ebps* 5 (20.8%), and the *fnbA*, *bbp* and *clfA* genes were not detected in any MRSA isolate. MLST analysis revealed 11 sequence types among MRSA isolates as follows: ST239, ST291, ST22, ST861, ST889, ST8, ST59, ST343, ST772, ST6 and ST1465. Also seven MLST-based clonal complexes (CCs) were identified among MRSA strains including: CC8, CC7, CC398, CC59, CC22, CC1 and CC5.

**Conclusions:**

A relatively high diversity was found in MRSA genotypes in Kashan and Isfahan hospitals, and seven clonal complexes were identified. Pandemic MRSA clones including CC8 and CC22 were the most prevalent clones and the novel ST types including ST1465, ST861, ST 889 and ST772 are reported for the first time in Iran in the present study. In addition the high prevalence of MSCRAMMs genes in MRSA isolates demonstrates the high potential of these strains for pathogenicity.

## Background

*Staphylococcus aureus (S. aureus)* is one of the most important human pathogen that causes a wide range of infections [1]. An important part of *S. aureus* success in developing the disease depends on its ability to bind to host cells and extracellular matrix proteins [2, 3]. Adherence to host cells and proteins is mediated by adhesive molecules which are called microbial surface components recognizing adhesive matrix molecules (MSCRAMMs) [4, 5]. These components are attached covalently to peptidoglycan by sortase enzymes [2, 6]. The bacterium is also highly regarded for its ability to obtain resistance to diverse antibiotics [2]. The emergence of methicillin-resistant *S. aureus* (MRSA) strains is an increasing public health challenge associated with high mortality and morbidity [7]. MRSA strain generates a low-affinity penicillin binding protein (PBP2a) that is responsible for the resistance to beta-lactam antibiotics [7]. PBP2a is encoded by *mecA* gene, carried by the Staphylococcal cassette chromosome *mec* (SCC*mec*) which as a large mobile genetic element is integrated to a region of the chromosome of MRSA [8]. In order to carry out epidemiological studies of MRSA strains several typing methods are available [9, 10]. Staphylococcal cassette chromosome *mec* (SCC*mec*) typing is a trustful typing method especially to make difference between hospital-acquired MRSA (HA-MRSA) and community-associated MRSA (CA-MRSA) [10]. Till now 13 SCC*mec* types have been documented and majority of MRSA strains which isolate from HA-MRSA strains bear SCC*mec* types I, II, and III [8]. Sequencing based typing methods such as multilocus sequence typing (MLST) have recently been introduced and received a lot of attention [10]. MLST typing is a reliable method to study genetic macro- variation in large populations based on specification of allelic profile of fragments of seven house-keeping genes [10–13].. There are some reports of molecular analysis and typing of MRSA strains from all over the world, although there is limited data regarding major MRSA clones circulating in our region. In the present study we used two reliable typing methods including MLST and SCC*mec* typing to reveal and compare the common genotypes of MRSA isolates circulating in four teaching hospitals in Isfahan and Kashan. Also the prevalence of virulence and MSCRAMMs genes among these MRSA strains was evaluated.

## Methods

### Bacterial strains and identification

A total of 146 *S. aureus* strains were isolated from clinical samples of patients admitted to teaching hospitals in Isfahan (*n* = 96) and Kashan (*n* = 50) during June 2017 to September 2018. Informed consent from all patients was obtained by survey questionnaire. The samples were cultured on blood agar (Merck, Germany), and plates were incubated at 37 °C for 24 h. *S. aureus* strains were identified by conventional microbiological methods including gram staining, and biochemical tests such as catalase, coagulase, mannitol fermentation, and DNase tests [14].

### Antimicrobial resistance patterns and phenotypic isolation of MRSA strains

The antimicrobial resistance patterns of *S. aureus* strains were performed by agar disc diffusion method according to the Clinical and Laboratory Standards Institute (CLSI) [15]. The antibiotic discs were prepared from MAST Company (MAST, UK) as follows: tetracycline (T; 30 μg), erythromycin (E; 15 μg), clindamycin (CD; 2 μg), cefazolin (CZ; 30 μg), cefoxitin (FOX; 30 μg), linezolid (LZD; 30 μg), ciprofloxacin (CIP; 5 μg), gentamicin (GEN; 10 μg), and trimethoprim sulfamethoxazole (TS; 25 μg). MICs for vancomycin were determined by broth microdilution methodology recommended by the CLSI [16]. The *S. aureus* strains ATCC 25923 and ATCC 29213 were used as quality control strains for agar disk diffusion and broth microdilution, respectively. For confirmation of MRSA strains, cefoxitin (30 μg) and oxacillin (1 μg) disks (Mast, UK) and disc diffusion method was performed on Mueller- Hinton agar (Merck, Germany) in accordance with the CLSI [16].

### DNA extraction and detection of *femA* and *mec*A genes

DNA extraction was performed by a standard phenol-chloroform method as previously described [1]*. All phenotypically isolated MRSA strains were confirmed by polymerase chain amplification (PCR),* for this purpose*, femA and mecA genes were amplified. For amplification of mecA gene* forward and reveres primers were used to detect a 268 bp fragment (Table 1) [19]. The PCR thermocycling program was: initial denaturation step at 97 °C for 6 min; followed by 30 cycles of 92 °C for 30 s, 55 °C for 30 s and 72 °C for 45 s, which finally ended with final extension step at 72 °C for ten min [20]. The *S. aureus* strain COL which carry *mecA* gene was used as positive control.

**Table 1:** The primers of genes used in this study

Target	Gene	Primer sequence (5′-3′)	Size of product (bp)	Reference
*femA*	*femA*	F: TGCTATCCACCCTCAAACAGG R: AACGTTGTAACCACCCCAAGA	450	[13]
*mecA*	*mecA*	F: TGCTATCCACCCTCAAACAGG R: AACGTTGTAACCACCCCAAGA	286	[17]
SCC*mec*	*ccrA2*	F: TAAAGGCATCAATGCACAAACACT R: AGCTCAAAAGCAAGCAATAGAAT	937	[18]
	*ccrC*	F: CCTTTATAGACTGGATTATTCAAAATAT R:CGTCTATTACAAGATGTTAAGGATAAT	518	
	*IS1272*	F: GCCACTCATAACATATGGAA R: CATCCGAGTGAAACCCAAA	415	
	*mecA-IS431*	F: TATACCAAACCCGACAACTAC R: CGGCTACAGTGATAACATCC	359	
MSCRAMMs genes	*bbp*	F: AACTACATCTAGTACTCAACAACAG R: ATGTGCTTGAATAACACCATCATCT	575	[5]
	*cna*	F: GTCAAGCAGTTATTAACACCAGAC R: AATCAGTAATTGCACTTTGTCCACTG	423	
	*eno*	F: ACGTGCAGCAGCTGACT R: CAACAGCATYCTTCAGTACCTTC	302	
	*ebpS*	F: CATCCAGAACCAATCGAAGAC R:CTTAACAGTTACATCATCATGTTTATCTTTG	186	
	*fnbA*	F: GTGAAGTTTTAGAAGGTGGAAAGATTAG R: GCTCTTGTAAGACCATTTTTCTTCAC	643	
	*fnbB*	F: GTAACAGCTAATGGTCGAATTGATACT R: CAAGTTCGATAGGAGTACTATGTTC	524	
	*fib*	F: CTACAACTACAATTGCCGTCAACAG R: GCTCTTGTAAGACCATTTTCTTCAC	404	
	*clfA*	F: ATTGGCGTGGCTTCAGTGCT R: CGTTTCTTCCGTAGTTGCATTTG	292	
Housekeeping genes	*arc*	up: TTGATTCACCAGCGCGTATTGTC dn: AGGTATCTGCTTCAATCAGCG	456	www.pubmlst.com
	*aro*	up: ATCGGAAATCCTATTTCACATTC dn: GGTGTTGTATTAATAACGATATC	456	
	*glp*	up: CTAGGAACTGCAATCTTAATCC dn: TGGTAAAATCGCATGTCCAATTC	465	
	*gmk*	up: ATCGTTTTATCGGGACCATC	429	
		dn: TCATTAACTACAACGTAATCGTA		
	*pta*	up: GTTAAAATCGTATTACCTGAAGG dn: GACCCTTTTGTTGAAAAGCTTAA	474	
	*tpi*	up: TCGTTCATTCTGAACGTCGTGAA dn: TTTGCACCTTCTAACAATTGTAC	402	
	*ygi*	up: CAGCATACAGGACACCTATTGGC dn: CGTTGAGGAATCGATACTGGAAC	516	

The 450 bp amplicon of *femA* gene was detected by PCR assays using specific primers and thermocycler (Eppendorf master cycler®, MA) (Table 1) with following run program: initial denaturation at 94 °C for 5 min, 40 cycles of 94 °C for 30 s, 55 °C for 40 s and 72 °C for 50 s and final extension at 72 °C for 10 min [17]. The PCR products were visualized after electrophoresis on 1% agarose gel under UV transilluminator (Bio-Rad, UK).

### Multiplex PCR for *SCCmec* typing

Five main *SCCmec* types of MRSA srtains were determined using multiplex PCR method with specific primers according to method previously presented by Boye et al. [18, 21]. For this purpose PCR reactions were performed in a final volume of 25 μL. Amplification was done with initial denaturation step (94 °C, 4 min), 30 cycles of denaturation (94 °C, 30 s), annealing (55 °C, 30 s), extension (72 °C, 60 s), and a final extension at 72 °C for 4 min, (Table 1).

### Identification of adhesive matrix molecules MSCRAMMs genes

The PCR amplification of MSCRAMMs genes were performed according to Tristan et al. [6] PCR1 was performed to amplify *bbp*, *cna*, *ebpS*, and *eno* genes and PCR2 was applied to amplify *fnbA*, *fnbB*, *fib*, and *clfA*, genes. The thermal cycling condition of multiplex PCR included an initial denaturation step (5 min at 94 °C) followed by 25 cycles of denaturation (1 min at 94 °C), annealing (1 min at 55 °C), and extension (1 min at 72 °C). The reaction was terminated with a 10 min incubation step at 72 °C [7]. (Table 1).

### Multilocus sequence typing (MLST)

The MLST types of 24 identified MRSA strains were determined. Identification of internal fragments of 7 housekeeping genes was performed by PCR amplification according to MLST websites (www.mlst.net) [22]. (Table 1). The final volume of PCR mixture was 25 μL with thermal program including: 95 °C for 15 min followed by 30 cycles of (95 °C for 1 min), (51 °C for 1 min), and (72 °C for 1 min) and finally 72 °C for 5 min [22].

### **Data a**nalysis

Data were analyzed using SPSS software Version 19.0. We were used Chi square or Fisher’s exact tests to determine the significance of the differences. A difference was considered statistically significant if the *P*-value was less than 0.05.

## Results

This study was performed on 146 clinical samples [Female = 82 (56.2%); Male = 64 (43.8%)] collected from teaching hospitals in Isfahan (Imam Musa Kazem, Amin and Al-Zahra Hospitals) and Kashan (Shahid Beheshti Hospital). Clinical samples were collected of burn wound 56 (38.4%), eye infection 49 (33.6%), respiratory infections 11 (7.5%), trauma 6 (4.1%), diabetic wound 5 (3.4%), brain abscess 5 (3.4%), blood 3(2%), urine 9(6.2), and other infections 2 (1.4%). There was no significant difference between MRSA and MSSA isolates in terms of age groups, gender and clinical specimens (*P* > 0.05), although a significant correlation was seen between the methicillin resistance and teaching hospitals from which clinical samples has been obtained (*P* = 0.001).

The results of PCR amplification of *mecA* gene showed among 146 studied *S. aureus* isolates 24 (16.4%) isolates identified as MRSA strains.

According to results obtained from antibiotic susceptibility testing, resistance rates to tetracycline 19 (79.2%), erythromycin 17 (70.8%), ciprofloxacin 16 (66.7%), and gentamicin 15 (62.5%) was high among MRSA strains and all *S. aureus* isolates were sensitive to vancomycin (MIC < 2 μg/mL) (Table 2). The MRSA strains were significantly more resistant to the antibiotics studied in comparison to MSSA strains (*P* < 0.05), and there was a significant correlation between multiple-drug resistance and MRSA isolation (*P* = 0.001).

**Table 2:** Antimicrobial resistance among MRSA and MSSA strains

Antibiotic (%)	MRSA (%) *N* = 24	MSSA (%) *N* = 122	Total (%) *N* = 146
Tetracycline	19 (79.2)	31(25.4)	50 (34.2)
Erythromycin	17 (70.8)	28 (22.9)	45 (30.8)
Ciprofloxacin	16 (66.7)	17 (13.9)	33 (22.6)
Gentamicin	15 (62.5)	9 (7.3)	24 (16.4)
Clindamycin	14 (58.3)	8 (6.6)	22 (15.1)
Cefazolin	13 (54.2)	1 (1)	14 (9.6)
Linezolid	1 (4.2)	2 (1.6)	3 (2.1)
Trimethoprim Sulfamethoxazole	8 (33.3)	6 (4.9)	14 (9.6)

### *SCCmec* typing

Three different SCC*mec* types were obtained among MRSA strains including 16 (66.7%) SCC*mec* type V, 3 (12.5%) SCC*mec* type III and, 5 (20.8%) SCC*mec* type II (Table 3).

**Table 3:** Characteristics of the MRSA isolates from clinical samples in different hospitals (*N* = 24)

Source	Hospital	Ward	Antibiotic resistance pattern	MSCRAMMs	SCCmec type	Clonal Complex	ST^*d*^
Blood	SB^*a*^	Emergency	T^*^, E, CIP, GEN, CD, CZ, TS, FOX	*eno, cna, fib, fnbB*	II	8	239
Burn wound	IMK^*b*^	Skin	T, E, CIP, GEN, CD, CZ, TS, FOX	*eno, cna, fib, fnbB*	III	8	861
	IMK	Skin	T, E, CIP, GEN, CD, CZ, FOX	*eno, cna, fib*	III	7	239
	IMK	Skin	E, CIP, GEN, CZ, FOX	*eno*	V	8	8
	IMK	Skin	T, CIP, LZD, FOX	*eno, ebp, fib, fnbB*	V	398	291
	IMK	Skin	T, E, CIP, GEN, CD, TS, FOX	*eno, fib*	V	59	59
	IMK	Skin	FOX	*eno, cna*	V	59	59
	IMK	Skin	T, FOX	_	II	398	291
	IMK	Skin	T, FOX	_	II	22	22
	IMK	Skin	T, E, CIP, GEN, CD, CZ, FOX	*eno, cna, ebp, fib*	II	8	239
	IMK	Skin	T, CZ, TS, FOX	*eno*	V	8	1465
	IMK	Skin	T, TS, FOX	*eno*	V	22	22
	IMK	Skin	E, FOX	*eno,cna, ebp, fib*	V	1	772
	IMK	Skin	T, E, CIP, GEN, CD, CZ, TS, FOX	*eno*	V	22	22
	IMK	Skin	E, FOX	*eno, ebp, fib*	V	5	6
	IMK	Skin	T, E, CIP, GEN, CD, CZ, TS, FOX	*eno, fib*	V	8	1465
Eye infection	SB	Emergency	T, E, CIP, GEN, CD, TS, FOX	*eno*	II	8	889
	Amin	Emergency	T, E, CIP, GEN, CD, CZ, FOX	*fib*	III	8	239
	Amin	Emergency	T, E, CIP, GEN, CD, CZ, FOX	_	V	8	1465
	Amin	Emergency	T, E, CIP, GEN, CD, CZ, FOX	*fib*	V	59	59
	Amin	Emergency	T, CIP, GEN, CD, CZ, FOX	_	V	22	22
	SB	Emergency	T, E, CIP, GEN, CD, CZ, FOX	*eno*	V	398	291
Brain abscess	AZ^*c*^	ICU	T, E, CIP, GEN, CD, FOX	*eno, ebp, fib, fnbB*	V	8	343
Respiratory infection	AZ	ICU	E, FOX	*fib*	V	22	22

^*a*^Imam Musa Kazem hospital; ^*b*^Shahid Beheshti; ^*c*^Al Zahra; ^*d*^Sequence type

^*^CIP ciprofloxacin, *CD* clindamycin, *CZ* cefazolin, *E* erythromycin, *FOX* cefoxitin, *GEN* gentamicin, *LZD* linezolid, *T* tetracycline, *TS* trimethoprim sulfamethoxazole

### Identification of MSCRAMMs genes

Of 24 MRSA isolates, 20 (83.3%) carried MSCRAMMs genes and in 4 (16.6%) of which, none of the MSCRAMMs genes studied were isolated. The prevalence of *eno*, *fib, cna, fnbB, ebps,* genes in MRSA isolates were 17 (70.8%), 13 (54.1%), 6 (25.0%), 4 (16.6%) and 5 (20.8%), respectively. Six, 2, and 4 isolates carried 4, 3 and 2 bands related to MSCRAMMs determinants respectively and the *fnbA*, *bbp* and *clfA* genes were not detected in any MRSA isolate (Table 3).

In statistical analyses a significant correlation was obtained between MRSA strains and *eno*, *cna* and *fib* genes, (*P* < 0.001). In statistical analyses a significant correlation was obtained between MRSA and MSSA strains regarding *eno*, *cna* and *fib* genes (*P* < 0.001).

### Multilocus sequence typing (MLST)

MLST analysis revealed 11 sequence types among MRSA isolates. The results of the MLST were as follows: ST239-SCC*mec* type III (2 isolates), ST239-SCC*mec* type II (2 isolates), ST291-SCC*mec* type V (2 isolates), ST291-SCC*mec* type II (1 isolates), ST22-SCC*mec* type II (1 isolate), ST22-SCC*mec* type V (4 isolates), ST861-SCC*mec* type III (1 isolate), ST 889-SCC*mec* type II (1 isolate), ST8-SCC*mec* type V (1 isolate), ST59-SCC*mec* type V (3 isolates), ST343-SCC*mec* type V (1 isolate), ST772-SCC*mec* type V (1 isolate), ST6-SCC*mec* type V (1 isolate) and ST1465-SCC*mec* type V (3 isolates). Also seven MLST- based clonal complexes (CCs) were identified among MRSA strains including: CC8 (41.7%), CC7 (4.2%), CC398 (12.5%), CC59 (12.5%), CC22 (20.7%), CC1 (4.2%) and CC5 (4.2%) (Table 3).

## Discussion

Nowadays, different clones of antibiotic-resistant strains, such as the MRSA strains, are spreading between healthcare centers and the community [13]. This issue is one of the major challenges of health care systems in the world [13]. The goal of this study was to determine the diversity of common clones, prevalence of virulence genes and antibiotic susceptibility patterns of MRSA strains in four University Teaching Hospitals in the center of Iran. It has been documented that MRSA clones are changing, and CA-MRSA are expanding into healthcare settings [23]. According to different previous studies, SCC*mec* types I-III and IV-V are commonly responsible for HA-MRSA and CA-MRSA infections, respectively [24]. In the present study, in contrast SCC*mec* type V was the most prevalent SCC*mec* type identified among our clinical isolates of MRSA. However recent studies conducted in Armenia and Iran, SCC*mec* types V and IV were the most types that identified among MRSA isolates from hospitals respectively [13, 25] Also in a study by Hallin et al. [26] in Belgium, most clinical MRSA strains belonged to SCC*mec* types IV. These results could confirm the rotation of clones between community and hospital. In MLST analysis of 24 MRSA isolates, 11 sequence types were identified and among them the ST22 and ST239 were most prevalent. All our MRSA strains with sequence type ST22 were isolated from hospitals in the city of Isfahan, and majority of them, belonged to SCC*mec* type V expect one isolate which was ST22-SCC*mec* type II. These MRSA ST22 types contained limited MSCRAMMs genes, although two isolates from burn wound and eye infection demonstrated multi-drug resistant (MDR) phenotype. In a study conducted by Goudarzi et al. [24] in the city of Tehran, ST22 has been documented as the third most commonly detected MRSA clone though the mentioned strains belonged to ST22-SCC*mec* IV in comparison to our MRSA ST22 which was ST22-SCC*mec* V. These findings highlight the importance of using several typing methods simultaneously to achieve better analysis. In previous studies of MRSA and MSSA strains in the cities of Isfahan and Tehran the majority of MRSA isolates belonged to ST239 [25]. The high prevalence of ST239 has been documented from many Asian countries especially India, Pakistan, Vietnam, Thailand, Taiwan, China, Sri Lanka, and Singapore [27]. In accordance with other studies, our MRSA isolates with ST239 belonged to SCC*mec* types II and III, with similar antimicrobial resistance and virulence patterns [22]. Another interesting founding was that these MRSA strains have been isolated from diverse clinical samples in different hospitals in Isfahan Province indicating circulation of these ST clones in clinical settings of our regains. During the time of current study, the third most commonly identified genotypes were ST59-SCC*mec* V, ST291-SCC*mec* V and ST1465-SCC*mec* V. The association of ST291 with HA- MRSA has been reported from Iran [28], and ST59 has been identified among both HA- MRSA and CA- MRSA strains in China [29]. Also ST59- SCC*mec* V has been documented as the predominant CA- MRSA strains in Asia [30]. However, ST1465, ST861, ST 889 and ST772 are reported for the first time in Iran in the present study. Our identified ST types belonged to seven clonal complexes, from which CC8 was the most predominant clone with MDR phenotype and was found in all the studied hospitals. The second most common clonal complex in the present study was CC22 which was not identified in Shahid Beheshti hospital in Kashan. According to MLST genotyping, *S. aureus* strains have been documented to be highly clonal and the most of the strains belong to a limited number of closely related genotypes. Literature have shown that CC8 (Iberian clone, Brazilian clone, Archaic clone), CC5 (New York/Japanese clone, Pediatric clone) and CC22 (epidemic MRSA-15) belong to epidemic MRSA (EMRSA) clones with global distribution in hospitals [27]. In accordance with our study, CC8-V was found as predominant clone in study conducted in Armenia [13]. Also genotyping methods have been showed that CC22 as the dominant clone are among major clones circulating in Tehran, Iran [21]. ST22 strains which are related to CC22 were determined as the predominant CA-MRSA clone in central of Iran [31]. Furthermore, the ST22 clone has previously been documented as a predominant CA-MRSA clone in Germany [32]. The increasing prevalence of the ST22 clone among HA-MRSA strains in recent studies represents that these strains may have been transferred from community to hospitals. Another interesting finding was that, of the new ST groups of MRSA found in current study, ST1465, ST861, and ST889, belonged to CC8. Among them, ST1465 and ST889 were isolated from eye infections and burn wounds in the cities of Isfahan and Kashan that indicates these clones are rotating in hospitals in our region. It is noteworthy that the mentioned strains had a limited number of MSCRAMMs genes studied (*eno*, *fib*) and none of these genes were found in one strain with ST1465. However, further studied is needed to give a precise information about the virulence of these strains. In addition the MRSA- ST861 strain was one of the most virulent strains studied, carrying four MSCRAMMs determinants including *eno*, *cna*, *fib*, *fnbB*. Since all of these strains had a MDR phenotype, they should be carefully considered. Finally, the MRSA-ST772 strain belonged to CC1, and found to carry *eno*, *cna*, *ebp*, and *fib* genes.

Our results confirmed that majority (83.3%) of the MRSA isolates harbored genes of binding factors and the prevalence of *eno* and *fib* genes encoding binding proteins was high. The high prevalence of adhesive protein genes in MRSA isolates indicates the persistence of these strains after colonization and subsequent infection. From the point of view of MSCRAMMs genes and correlation with ST groups, the most virulent strains of our MRSA isolates were belonged to CC8, CC1, and CC398. The *eno* and *fib* genes were present in all seven identified clonal complexes, whereas the *cna* was detected in the CC1, CC7, CC8, CC59, and the *ebp* gene identified in the CC1, CC5, CC8, and CC398. The *fnbB* gene, encoding fibronectin binding protein B was detected in only two clonal complexes including CC8 and CC398. However, all isolates that carried the *fnbB* gene were identified as the most virulent strains with multiple resistance phenotypes and isolated from various hospitals and important clinical samples including blood and brain abscess. In other studies, all of the *S. aureus* isolates from the bloodstream have been showed to carry the *fnb* gene [3]. Since CC8 is the most dominant clone in our region, the presence of the mentioned virulence genes in this clone is of great importance.

## Conclusion

According to MLST and SCC*mec* typing results, a relatively high diversity was found in MRSA genotypes in the hospitals of Kashan and Isfahan, and seven clonal complexes were identified. However, it should be considered that only a limited number of isolates were genotyped during current study. Pandemic MRSA clones including CC8 and CC22 were the most prevalent clones and the novel ST types including ST1465, ST861, ST 889 and ST772 are reported in the present study for the first time in Iran. Furthermore, presence of momentous sequence types including ST22-SCC*mec* type II, ST239-SCC*mec* types II and III and ST291-SCC*mec* type II among important clinical samples such as burn wound and blood due to the risk of further spread of these global multi-antibiotic resistant strains is noteworthy. In addition the high prevalence of MSCRAMMs genes in MRSA isolates demonstrates the high potential of these strains for pathogenicity.

## Data Availability

The data analyzed during the study will be available from the corresponding authors upon request.
